# Metabolic Dysfunction-Associated Steatotic Liver Disease: A Silent Driver of Cardiovascular Risk and a New Target for Intervention

**DOI:** 10.3390/ijms26168081

**Published:** 2025-08-21

**Authors:** Giovanna Gallo, Gabriele Nalli, Francesco Baratta, Giovambattista Desideri, Carmine Savoia

**Affiliations:** 1 Department of Clinical and Molecular Medicine, Faculty of Medicine and Psychology, Sapienza University of Rome, Via di Grottarossa 1035–1039, 00189 Rome, Italy; giovanna.gallo@uniroma1.it (G.G.); gabriele.nalli@uniroma1.it (G.N.); 2 Geriatric Unit, Department of Internal Medicine and Medical Specialties, AOU Policlinico Umberto I, 00161 Rome, Italy; francesco.baratta@uniroma1.it; 3 Department of Medical and Cardiovascular Sciences, Sapienza University of Rome, Viale del Policlinico 155, 00161 Rome, Italy; giovambattista.desideri@uniroma1.it

**Keywords:** MASLD, MASH, cardiometabolic risk, cardiovascular disease, GLP-1 agonists, GIP-1

## Abstract

Metabolic dysfunction-associated steatotic liver disease (MASLD) significantly increases the risk of steatohepatitis and cirrhosis and multiple extrahepatic complications, in particular, cardiometabolic disease, including type 2 diabetes, atherosclerotic cardiovascular disease (CVD), and heart failure, with a significant negative impact on health-related quality of life, becoming a substantial economic burden. Moreover, cardiovascular events represent the leading cause of death in MASLD patients. A timely diagnosis stratifies patient for their risk. It can facilitate early lifestyle changes or pharmacological management of dysmetabolic conditions, thereby slowing disease progression, lowering cardiovascular risk, and preventing CVD and cirrhosis. In this narrative review, we will discuss the current knowledge on MASLD and metabolic dysfunction-associated steatohepatitis (MASH) pathophysiology, emphasizing their systemic nature, the link to CVD, and available and emerging treatment strategies.

## 1. Introduction

Metabolic dysfunction-associated steatotic liver disease (MASLD) is defined by the presence of hepatic steatosis in association with metabolic dysfunction. It has emerged as the most prevalent form of chronic liver disease worldwide, affecting over 38% of the adult population and approximately 7–14% of children and adolescents [1]. These rates are much higher in people with type 2 diabetes mellitus (T2DM), with an estimated prevalence of 70% for MASLD [2], with approximately half having the more progressive form with metabolic dysfunction–associated steatohepatitis (MASH) and about one in five having advanced liver fibrosis [3,4,5].

MASH is histologically characterized by lobular inflammation, hepatocyte ballooning, and varying degrees of fibrosis, which may evolve into steatohepatitis, cirrhosis, and hepatocellular carcinoma (HCC) [6] with increased overall mortality. Approximately 20–30% of individuals with MASLD develop MASH, and 15–20% of MASH patients ultimately progress to cirrhosis [6].

MASLD is the most recent term, adopted in 2023, to define this disease. In 2020, metabolic dysfunction-associated fatty liver disease (MAFLD) was introduced to better include metabolic risk factors in patients with coexisting liver conditions, replacing the term nonalcoholic fatty liver disease (NAFLD), to reflect its pathogenesis better as a liver disorder strongly rooted in metabolic dysfunction rather than defined by the absence of alcohol intake. Thereafter, the proposed use of the MASLD term focuses more on better metabolic profiles and could support a more integrated approach, enhancing management and outcomes for patients with hepatic metabolic comorbidities and improving clinical outcomes. Similarly, regarding the term NASH (nonalcoholic steatohepatitis), it was decided to introduce the acronym MASH to emphasize the importance of inflammation associated with excessive hepatic lipid accumulation and better relate this condition to metabolic dysfunction [7].

The diagnostic criteria for MASLD require evidence of hepatic steatosis in conjunction with at least one cardiometabolic risk factor, including overweight/obesity, T2DM, insulin resistance, dyslipidemia, or hypertension (Table 1).

MASLD is not limited to liver-related outcomes. A large body of evidence has demonstrated that MASLD significantly increases the risk of multiple extrahepatic complications, particularly cardiometabolic diseases, including type 2 diabetes, atherosclerotic cardiovascular diseases (ACVD), heart failure, and extrahepatic malignancies [5], with a significant negative impact on health-related quality of life becoming a substantial economic burden [8]. In large cohort studies, patients with MASLD had a 2-fold increased risk of cardiovascular mortality and morbidity, independent of traditional cardiovascular disease (CVD) risk factors [9].

An early diagnosis is possible by using noninvasive tests (NITs) to stratify patients for their risk of developing CVD and cirrhosis and can facilitate an adequate pharmacological and non-pharmacological management, thereby slowing disease progression, lowering cardiovascular risk, and preventing CVD and cirrhosis.

Despite this evidence, most individuals and their healthcare professionals remain unaware of the severe hepatic or extrahepatic health risks associated with MASLD and the need for early identification. Thus, given the high burden of cardiovascular complications and the limitations of current awareness, there is a pressing need to clarify the underlying mechanisms linking MASLD to extrahepatic outcomes (particularly in the cardiovascular system) and to explore emerging therapeutic avenues.

Several reviews have examined the association between MASLD (formerly NAFLD/MAFLD) and cardiovascular risk [1]. Still, most have focused either on hepatic outcomes with only a secondary mention of CVD [9,10,11,12,13,14] or on limited aspects of cardiometabolic comorbidities without integrating mechanistic, diagnostic, and therapeutic perspectives [10,15,16]. Moreover, few have incorporated the recent nomenclature change to MASLD/MASH [1,2,3,4] and the rapidly emerging evidence from the past 2–3 years, including noninvasive cardiovascular risk assessment tools, the role of gut–liver–heart crosstalk and novel pharmacotherapies developed initially for diabetes and cardiovascular disease.

In this narrative review, we will discuss the current knowledge on the MASLD and MASH pathophysiology, emphasizing their systemic nature, particularly the link to CVD risk. We will highlight the clinical implications of explicitly framing MASLD/MASH as subclinical multiorgan damage and a modifiable cardiovascular risk factor and discuss available and emerging treatment strategies.

### Methodological Aspects, Literature Search Strategy, and Framework for Evidence Synthesis

This narrative review was conducted through a comprehensive literature search on the current evidence on MASLD and MASH as cardiometabolic risk factors, focusing on their cardiovascular implications and therapeutic perspectives.

PubMed/MEDLINE, Embase, Scopus, and the Cochrane Library were systematically searched up to July 2025. Search terms included combinations of the following keywords and Medical Subject Headings (MeSH): “metabolic dysfunction-associated steatotic liver disease”, “MASLD”, “metabolic dysfunction-associated steatohepatitis”, “MASH”, “nonalcoholic fatty liver disease”, “NAFLD”, “cardiovascular risk”, “cardiometabolic disease”, “heart failure”, “atherosclerosis”, “arrhythmia”, “diabetes”, “GLP-1 receptor agonists”, “SGLT2 inhibitors”, and “treatment”. Boolean operators (“AND”; “OR”) were applied to combine terms. Reference lists of included articles and relevant reviews were also screened to identify additional studies.

Original research articles, systematic reviews, meta-analyses, clinical guidelines, and consensus statements were selected. Moreover, human and preclinical studies were selected with additional key mechanistic insights from relevant experimental research and studies addressing epidemiology, pathophysiology, mechanisms, diagnostic assessment, cardiovascular outcomes, or therapeutic interventions in MASLD/MASH as cardiovascular risk factors.

Priority was given to high-quality evidence from preclinical and clinical studies, randomized controlled trials, extensive prospective cohort studies, and recent meta-analyses. When available, guideline and consensus recommendations from major societies (e.g., EASL, AASLD, ADA, and ESC) were integrated to contextualize findings.

The evidence was synthesized narratively, structured around the following domains: (1) epidemiology and disease definitions and evolution; (2) pathophysiological mechanisms linking MASLD/MASH to cardiovascular risk; (3) clinical and subclinical cardiovascular manifestations; (4) diagnostic and risk assessment tools; and (5) therapeutic strategies, including lifestyle interventions, pharmacologic agents, and emerging therapies.

## 2. MASLD and MASH Characteristics and Evolution

MASLD has emerged as the most prevalent cause of chronic liver disease. The pathogenesis of MASLD and MASH is complex and results from interactions of genetic and environmental factors [10,11,12,17]. MASLD is characterized by excessive triglyceride accumulation in hepatocytes [13]. MASLD spans from simple hepatic steatosis, often with minimal inflammation or hepatocyte ballooning, to steatohepatitis (MASH), which involves marked hepatocellular injury and inflammation. With disease progression, MASLD may lead to fibrosis, cirrhosis, and eventually hepatocellular carcinoma (HCC) [14]. Long-term studies show that 12–40% of MASLD cases advance to MASH within 8–13 years [14,15,16], and about 15% of patients with early MASH-related fibrosis develop cirrhosis or hepatic decompensation, rising to 25% in those with advanced fibrosis (Table 2).

Although MASLD was previously thought not to cause progressive fibrosis [18], newer evidence suggests fibrosis can develop even in MASLD without MASH [19]. Around 18% of patients developed advanced fibrosis within six years, regardless of MASH status. This highlights the dynamic and potentially bidirectional nature of MASLD-MASH progression [20]. A meta-analysis of 11 such studies [21] found that fibrosis progression occurred in 39% of MASLD patients over 14 years, while 53% remained stable and 8% improved. In MASH, 35% progressed, 39% remained stable, and 27% improved. The average time to progress one fibrosis stage was 14 years in MASLD without baseline fibrosis, compared to 7 years in MASH patients.

Approximately 10–25% of MASH patients progress to advanced fibrosis or cirrhosis [15,16,18]. Over 4.5 years, 14% and 2% of those with stage 0–2 fibrosis advance to stages 3 and 4, respectively [22]. However, current data, mainly from small studies using paired liver biopsies, may overestimate progression rates, as they often involve selected high-risk patients rather than the general MASLD population.

Lack of fibrosis regression in compensated cirrhosis is linked to a nearly 10-fold increased risk of liver-related complications [23]. A prospective study found hepatic decompensation rates of 0.05/100PY in stage 0–2, rising to 0.99/100PY (HR:18.7) and 2.7/100PY (HR:36.1) in stages 3 and 4, respectively. New decompensation was strongly associated with all-cause mortality (HR:6.8) [24]. In the Simtuzumab trials, MASLD patients with hepatic venous pressure gradient (HVPG) ≥ 10 mmHg had a 3-fold higher risk of complications, with a 15% increase per 1 mmHg HVPG elevation [23]. Compared to HBV-related cirrhosis, MASH-related decompensated cirrhosis showed worse outcomes and higher healthcare use [25,26], and MASH cirrhosis has become the fastest-growing indication for liver transplantation in Western countries [27].

MASLD/MASH is now the leading cause of HCC in the USA (59% of cases) [28], and the fastest-growing HCC cause among transplant candidates [29]. Annual HCC incidence in Japanese MASLD patients mirrors USA rates (0.043–0.0627%) [30]. Around 7% of MASLD patients with compensated cirrhosis develop HCC within 10 years, and 50% of these require liver transplantation or die from liver-related causes. Interestingly, HCC can also occur without cirrhosis. Only 46% of MASLD-HCC patients had cirrhosis, versus 78% with HCV-HCC [28]. Obesity, T2DM, and patatin-like phospholipase domain-containing protein 3 (PNPLA3) variants (≥3-fold HCC risk) are key contributors [28,31,32,33,34,35]. Despite the link between MASLD and HCC, the cost-effectiveness of HCC surveillance in non-cirrhotic MASLD is still debated due to potential over-testing and false positives [36,37].

## 3. MASLD, MASH and Cardiometabolic Diseases

Beyond liver complications, MASLD is closely tied to insulin resistance, metabolic syndrome (MS), T2DM, obesity, and dyslipidemia and has shown stronger associations with overall and CV mortality in cohort studies [38,39,40,41]. One study found MASLD independently predicted all-cause mortality, even after adjusting for other metabolic risk factors [42]. Patients with MASH, advanced fibrosis, or MASLD with T2DM are considered high-risk for CVD. National Health and Nutrition Examination Survey (NHANES) data also identified MASLD in adults aged 45–54 as an independent predictor of CV mortality [43]. MASLD patients have a higher prevalence and incidence of CVD than matched controls, driven by shared cardiometabolic risk factors, comorbidities, and ectopic fat accumulation [44]. Atherosclerotic CVD is the leading cause of death in MASLD [45]. A meta-analysis of 16 studies (34,043 patients, 7-year median follow-up) found MASLD was linked to a 64% increased risk of fatal/nonfatal CV events, rising to 160% in advanced liver disease (F3–F4) [46]. Another meta-analysis showed that MASLD doubled CVD risk in T2DM patients [47].

However, while cardiometabolic risk factors play a significant role in linking MASLD to CVD, they may not fully explain this association. Therefore, the impact of MASLD on CVD risk remains still under investigation. Nonetheless, comprehensive cardiovascular risk assessment is strongly recommended in all MASLD patients [48,49].

### 3.1. Pathophysiological Aspects

#### 3.1.1. Insulin Resistance and Lipid Metabolism Abnormalities

**Figure 1:**
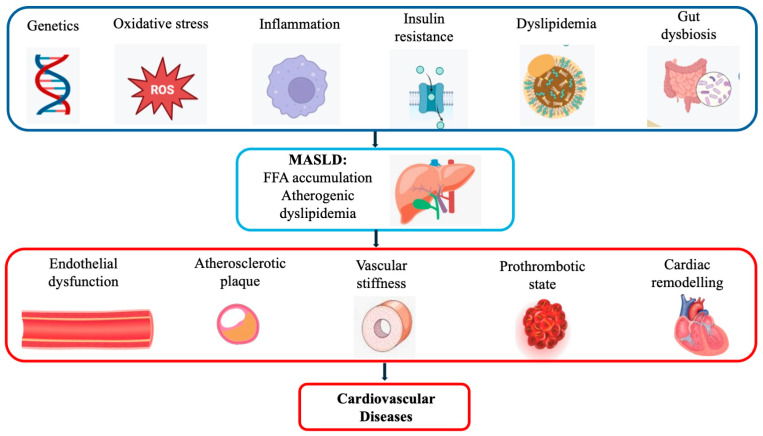
MASLD pathophysiology and clinical outcome. FFA, free fatty acid accumulation.

Insulin resistance is considered a central mechanism in the pathogenesis of MASLD. Commonly linked to obesity, MS, T2DM, and dysregulated lipid metabolism, insulin resistance promotes hepatic steatosis by increasing the influx of free fatty acids (FFAs) from adipose tissue [50,51]. Nonetheless, insulin resistance is also observed in individuals who are neither overweight nor diabetic, indicating that diverse pathogenic mechanisms may contribute to MASLD development [52]. As a key driver of both hepatic and cardiovascular disease, insulin resistance substantially increases the risk of myocardial infarction, stroke, and other cardiovascular events, even in the absence of diabetes (Figure 1).

Insulin resistance plays a pivotal role in the development and progression of lipid metabolism abnormalities in MASLD. FFAs originate from spontaneous lipolysis of adipose tissue, de novo lipogenesis, and excessive dietary intake, contributing to about 60%, 25%, and 15% of the total influx of FFAs to the liver, respectively [53,54]. Normally, the liver converts FFAs into triglycerides for export as VLDL, but insulin resistance disrupts this process, leading to lipid buildup in hepatocytes [50]. The imbalance between lipid input and output leads to excessive FFA accumulation in the liver, lipotoxicity, mitochondrial and endoplasmic reticulum dysfunction, excessive production of reactive oxygen species (ROS), and finally to the disruption of metabolic homeostasis [55,56]. In particular, increased glycolysis and FFA oxidation, with excessive acetyl-CoA production and enhanced tricarboxylic acid cycle (TCA) activity, promote ROS production and hepatocellular injury [57]. In addition, ketogenesis and mitochondrial respiratory chain activity reduction occur, along with a maladaptive expression of unfolded protein response (UPR) and the activation of inflammatory pathways [58], further amplifying tissue damage. The UPR contributes to keeping the endoplasmic reticulum (ER) balanced and functioning properly. But if activated too long, it can switch from protective to harmful, setting off cell death pathways that damage hepatocytes [59]. Under different stressful conditions, molecular chaperones undergo ectopic translocation and activate the immunoglobulin-regulated enhancer 1 (IRE1), protein kinase RNA-like endoplasmic reticulum kinase (PERK), and activating transcription factor 6 (ATF6) signaling pathways, which promote the synthesis of C/EBP-homologous protein (CHOP) and directly affect ER stress [60].

FFA accumulation also contributes to altered autophagic protective mechanisms in MASLD due to defective lysosomal acidification and lysosomal calcium retention [61]. FFA contributes to the translocation of Bax to lysosomes in hepatocytes, to the release of cathepsin B (ctsb) into the cytoplasm, and, finally, to lipotoxicity [62]. In addition, cholesterol from dead hepatocytes induces macrophage lysosomal dysfunction, promoting the progression to MASH [63].

Insulin resistance contributes to reducing the activity of lipoprotein lipase (LPL) and hepatic lipase, increasing plasmatic concentrations of atherogenic lipoproteins, such as small dense low-density lipoproteins (sdLDLs) and very low density lipoproteins (VLDLs) [55,64]. These lipid abnormalities play a central role in promoting atherosclerosis and CVD. SdLDL particles more easily penetrate the endothelium and become oxidized (ox-LDL), triggering inflammation and plaque formation, while their poor clearance enhances atherogenicity [65]. Low HDL levels also impair reverse cholesterol transport and reduce antioxidant and anti-inflammatory protection. This atherogenic dyslipidemia, along with hypertension, inflammation, and hyperglycemia, increases the risk of atherosclerotic cardiovascular disease (ASCVD) [66], underscoring the importance of lipoprotein metabolism in MASLD-related vascular disease.

#### 3.1.2. Type 2 Diabetes Mellitus and Obesity

T2DM and MASLD share a bidirectional relationship, since MASLD increases the risk of T2DM, while T2DM promotes MASLD onset and fibrosis progression [67,68]. MASLD patients are characterized by low response to insulin, the loss of the negative feedback on gluconeogenesis, and elevated blood glucose levels. The presence of constant low-grade systemic inflammation may contribute to increased pancreatic oxidative stress and accelerated development and progression of diabetes [69]. MASLD affects 42–70% of T2DM patients, and T2DM is found in 23% of MASLD cases, with prevalence rising alongside disease severity [67,70]. Patients with both conditions show higher fasting insulin and more pronounced insulin resistance than those with T2DM alone [71]. Additionally, a recent study found MASLD in about 20% of type 1 diabetes patients, linked to similar risk factors, like insulin resistance [72].

T2DM is strongly associated with the development of liver fibrosis and hepatocellular carcinoma in MASLD and significantly raises the risk of all-cause mortality and CVD [73,74]. Therefore, early identification and management of cardiovascular risk are essential, particularly in MASLD patients with T2DM. MASLD patients with T2DM, and even those without diabetes but with obesity or other metabolic risk factors, tend to experience worse clinical outcomes [75].

MASLD also has a strong relationship with obesity, especially central (abdominal) obesity and vascular adiposity, with an increased risk of evolution into liver fibrosis and MASH [76]. Obesity contributes to liver fat accumulation, inflammation, and cell damage, key features in MASLD development [77]. Thus, obesity plays a central role in both the onset of hepatic steatosis and progression to more severe liver damage [77]. Weight changes of ±5 kg are linked to corresponding shifts in disease activity and fibrosis scores [78]. As a major cardiovascular risk factor and core component of metabolic syndrome, obesity significantly heightens CVD risk when coexisting with MASLD. Addressing obesity can improve liver health and reduce cardiovascular complications [79,80].

However, available data indicate that about half of MASLD patients are nonobese, with this group showing higher all-cause mortality than their obese counterparts [81]. Lean MASLD accounts for 5.1% to 11.2% of cases globally, with the highest prevalence in Asia [82]. Lean MASLD patients typically have fewer metabolic comorbidities and similar *PNPLA3* and *TM6SF2* (transmembrane 6, superfamily member 2) variant frequencies compared to obese MASLD patients [83]. However, long-term studies suggest lean MASLD may be associated with higher rates of liver-related complications and mortality [84,85]. Nonetheless, obesity is still one of the main factors linking MASLD to cardiovascular CVD, making weight control essential for better health outcomes.

In MASLD, fat can accumulate in organs outside fat tissue, including the heart, pancreas, and muscles, disrupting normal metabolism [86,87]. The heart is especially affected, as this fat buildup can lead to damage through oxidative stress, alteration of mitochondria function, and chronic inflammation, resulting in structural changes, irregular heart rhythms, and reduced blood flow [86].

Studies have shown that higher levels of fat around the heart (pericardial fat) are linked to heart failure, especially in women [88], and fat around the aorta (periaortic fat) is associated with coronary artery disease, even in people who are not obese [89]. These findings show that ectopic fat plays a major role in heart risk for people with MASLD and should be taken into account when assessing and treating patients [90].

Visceral fat expansion, adipocyte hypertrophy, and macrophage infiltration impair insulin sensitivity and trigger inflammation, and alter adipokine secretion (↑leptin, ↓adiponectin) and lipotoxic lipid accumulation, contributing to disease progression in both lean and obese individuals [91,92,93].

Adipokine imbalance is a key contributor to MASLD, as it interferes with metabolic processes and drives inflammation. Adiponectin exerts anti-inflammatory effects by inhibiting the expression of proinflammatory cytokines in liver tissue and reducing hepatocellular injury and inflammatory cell infiltration [94,95]. Moreover, adiponectin enhances hepatic insulin sensitivity and inhibits gluconeogenesis and lipogenesis through activation of AMP-activated protein kinase (AMPK) and peroxisome proliferator-activated receptor-alpha (PPAR-α) pathways, which promote fatty acid oxidation. Adiponectin is commonly reduced in MASLD, promoting insulin resistance, liver fat buildup, and a higher risk of cardiovascular disease [96]. In particular, patients with hepatic steatosis, especially those progressing to steatohepatitis and fibrosis, show reduced circulating adiponectin concentrations [94,95].

In contrast, leptin levels are elevated and linked to liver fibrosis, inflammation, and atherosclerosis through proinflammatory and oxidative mechanisms [97,98]. Leptin enhances the production of proinflammatory cytokines (e.g., TNF-α, IL-6, and IL-12) in Kupffer cells, the liver-resident macrophages; promotes Th1 responses over Th2, favoring the release of IFN-γ and perpetuating inflammatory cascades; inhibits regulatory T cells (Tregs); and upregulates adhesion molecules (e.g., ICAM-1 and VCAM-1) on hepatic sinusoidal endothelial cells, facilitating leukocyte recruitment and transmigration into hepatic tissue [99,100,101].

Other adipokines, including resistin, visfatin, and chemerin, are also dysregulated in MASLD, contributing to insulin resistance and metabolic dysfunction [102], and ultimately to liver disease progression and a rise in the risk of type 2 diabetes and cardiovascular disease. This evidence underscores the pivotal role of adipose tissue in MASLD pathophysiology.

On the other hand, incretins, particularly glucagon-like peptide-1 (GLP-1), have emerged as important regulators of liver metabolism and are increasingly recognized for their role in MASLD pathophysiology and its progression to MASH. GLP-1 acts on hepatocytes through indirect and direct mechanisms, ultimately improving liver function and slowing disease progression [103,104]. Among its key indirect effects, GLP-1 promotes weight loss and appetite suppression, reducing adiposity and the influx of FFAs to the liver, lowering hepatic fat accumulation. It also enhances insulin sensitivity by stimulating glucose-dependent insulin secretion and suppressing glucagon release, improving glycemic control and reducing hepatic de novo lipogenesis [105].

#### 3.1.3. Inflammation and Endothelial Dysfunction

Systemic inflammation plays a crucial role in the development and progression of MASLD and its complications [106]. Rather than being confined to the liver, this inflammatory response has a systemic effect, particularly on the cardiovascular system [107,108]. MASLD is commonly associated with elevated levels of key proinflammatory cytokines, especially tumor necrosis factor-alpha (TNF-α) and interleukin-6 (IL-6). TNF-α promotes hepatic inflammation, insulin resistance, and steatosis, partly through activation of the NF-κB signaling pathway and stimulation of foam cell formation, key mechanisms in the pathogenesis of atherosclerosis [109,110]. IL-6 activates the JAK/STAT pathway, leading to increased production of acute-phase proteins such as C-reactive protein (CRP) and serum amyloid A (SAA), which further amplify both hepatic and systemic inflammation [111,112,113].

Visceral obesity releases different pro-inflammatory cytokines, contributing to insulin resistance, fat influx, mitochondrial dysfunction, hepatocellular injury, endothelial injury, and pro-atherogenic processes [114]. TNF-α, IL-6, IL-1β, and IL-18 exacerbate endothelial dysfunction by inhibiting nitric oxide (NO) synthesis and inducing the expression of vascular cell adhesion molecules, promoting leukocyte adherence, vascular inflammation, and plaque formation [115]. Furthermore, monocyte chemoattractant protein-1(MCP-1) and C-X-C motif chemokine ligands facilitate the recruitment of monocytes to the endothelium and their transformation into macrophages and foam cells, leading to the progression of atherosclerotic plaques [116].

Oxidative stress, arising from an imbalance between ROS and antioxidant defenses, also plays a pivotal role in the pathogenesis of MASLD and CVD [110]. In MASLD, hepatic lipid accumulation enhances ROS production, resulting in cellular injury, inflammation, fibrosis, and worsened insulin resistance [117]. Oxidative stress contributes to atherosclerosis in the cardiovascular system by damaging the endothelium, setting off inflammatory responses, and causing lipid oxidation, all speeding up plaque formation [118]. It also impairs heart and blood vessel function, raising the risk of hypertension, coronary artery disease, and heart failure [119]. Obesity, a common denominator in both MASLD and CVD, further exacerbates oxidative stress by elevating levels of VLDL, low-density lipoproteins (LDLs), and lipid peroxidation, thereby amplifying cardiovascular risk [81,120]. The chronic low-level inflammation impairs the endothelium, makes atherosclerotic plaques more prone to rupture, and damages blood vessels, altogether speeding up the development of atherosclerosis and increasing the risk of coronary artery disease [121,122].

Endothelial dysfunction is a common feature of ASCVD development and is closely associated with MASLD. The endothelium regulates vascular homeostatic mechanisms, including vascular tone, blood flow, and coagulation, primarily through vasodilators like NO [123,124]. Vascular function is impaired in MASLD patients due to increased oxidative stress and inflammation, leading to an increased CVD risk [125,126]. Moreover, elevated levels of asymmetric dimethylarginine (ADMA), an inhibitor of endothelial NO synthase (eNOS), are common in MASLD and further suppress NO production, leading to increased vascular resistance and blood pressure [127]. This is compounded by excess endothelin-1 (ET-1), a potent vasoconstrictor overproduced in MASLD under proinflammatory and oxidative conditions, promoting vascular smooth muscle proliferation and atherosclerosis [128]. Together, ADMA and ET-1 drive endothelial dysfunction, highlighting their role as therapeutic targets in reducing CVD risk in MASLD.

MASLD increases the risk of cardiovascular and vascular events by also promoting a hypercoagulable state. Elevated levels of coagulation factors (e.g., factor VIII, fibrinogen, and von Willebrand factor) and impaired fibrinolysis due to high plasminogen activator inhibitor-1 (PAI-1) contribute to clot formation [129,130]. Insulin resistance and inflammation enhance platelet reactivity and damage the endothelium, while reduced NO and increased adhesion molecules further promote thrombosis. These changes collectively heighten the risk of thromboembolic events such as myocardial infarction, stroke, and peripheral artery disease [123,131].

#### 3.1.4. Role of Gut Microbiota and Diet

Growing evidence highlights multiple mechanisms through which gut microbiota contributes to MASLD progression [132]. Gut dysbiosis, consisting of bacterial translocation from the small intestine and a change in bacterial composition, has also emerged as a mechanism involved in developing MASLD and contributing CVD development by promoting inflammation, increasing intestinal permeability, and affecting host metabolism and immune responses, through the production of inflammatory factors and the release of systemically absorbed toxins [71]. Dysbiosis is associated with a weakening of the intestinal barrier; a breakdown of the mucus layer; and a slippage of components of gut bacteria, such as lipopolysaccharides (LPS) from Gram-negative bacteria, or fragments like peptidoglycans and toxins like LPS through the gut wall into the bloodstream. These microbial products are recognized as threats by the immune system, particularly by innate immune cells, like macrophages and dendritic cells, generating the production of cytokines such as TNF-α, IL-6, and IL-1β and leading to a low-grade chronic systemic inflammation [72,132].

At the same time, dysbiosis often leads to a loss of beneficial bacteria, including *Faecalibacterium prausnitzii* and *Akkermansia muciniphila*, which produce short-chain fatty acids (SCFAs), such as butyrate, with protective effects for gut health, nourishing intestinal cells, strengthening the barrier, and even promoting the development of regulatory immune cells that help to prevent inflammatory over-reactions [133].

MASH patients often show reduced Bacteroidetes, lower *Prevotella*, and increased *Clostridium coccoides* levels [134,135]. Gut dysbiosis may explain increased intestinal permeability due to disrupted tight junctions in MASLD [134], allowing bacterial translocation and proinflammatory stimuli that activate stellate cells and promote fibrosis [135]. Endotoxins may also enhance leptin sensitivity, further driving fibrosis [10].

Gut microbiota influences bile acid (BA) composition, which plays a role in MASLD pathogenesis [134]. MASH patients display higher total fecal BA, increased synthesis, and altered BA ratios, indicating enhanced enterohepatic circulation [136,137]. BAs activate the Farnesoid X receptor (FXR), which regulates BA synthesis and glucose metabolism via FGF15/19 signaling in hepatocytes [138]. In mice, gut microbiota alterations affect taurine-conjugated BA, suppress FXR, and induce metabolic disorders like obesity and insulin resistance [139]. Therapeutic strategies targeting FXR (e.g., obeticholic acid) and peroxisome proliferator-activated receptors (PPARs) are under investigation for MASLD treatment [140].

Excess caloric intake, along with an unhealthy diet high in fructose, fat, and cholesterol, can accelerate MASLD progression [141] and contribute to ASCVD. High-fat intake contributes more to MASH–HCC development than obesity itself, indicating the diet’s role in liver cancer regardless of weight status [142]. Poor diet worsens MASLD’s metabolic profile, while the Mediterranean diet and moderate coffee consumption may offer protection to the liver and cardiovascular system [143,144]. Although heavy alcohol use is a known liver disease risk, even moderate consumption has been linked to advanced fibrosis after adjusting for metabolic factors [145,146]. As a result, complete alcohol abstinence is recommended for MASLD patients [147].

#### 3.1.5. Role of the Genetic Component

Growing evidence supports a significant genetic component in MASLD development. Multi-ethnic cohort studies show varying susceptibility among ethnic groups, with Hispanics at highest risk, Europeans intermediate, and African Americans lowest, even after adjusting for factors like body weight, T2DM, and socioeconomic status [148]. First-degree relatives of individuals with MASLD cirrhosis also face a higher risk, independent of confounders [149]. Different single-nucleotide polymorphisms (SNPs) in genes involved in insulin resistance (insulin receptor substrate-1, IRS1), retinol metabolism (patatin-like phospholipase domain-containing 3, *PNPLA3*), lipid transport (transmembrane 6 superfamily member 2, TM6SF2), and oxidative stress regulation (membrane-bound O-acyl-transferase domain-containing 7, *MBOAT7-TMC4*) have been described in patients with MASLD [150,151,152]. Genome-wide association studies (GWASs) have identified key genetic variants linked to MASLD progression, particularly in *PNPLA3*, *TM6SF2*, and *HSD17B13* [153]. Variants in *PNPLA3* and *TM6SF2* increase susceptibility to the full spectrum of MASLD-related liver damage [154]. In contrast, the *PNPLA3* I148M variant is associated with higher liver-related mortality in both MASLD patients and the general population [155]. The SNP rs738409 in the *PNPLA3* gene, resulting in the I148M variant, is strongly associated with increased liver fat, fibrosis, cirrhosis, and hepatocellular carcinoma, independent of traditional risk factors [156,157]. Although its direct impact on CVD is unclear, it may contribute to atherogenic dyslipidemia and elevated CVD risk [158]. Other variants, such as *TM6SF2 E167K* and *MBOAT7*, are linked to MASLD progression, with *TM6SF2* showing a paradoxical protective effect against cardiovascular events despite worsening liver disease [159,160]. Additional polymorphisms in GCKR and SREBP-1, which are involved in lipid metabolism, may also affect CVD risk [161]. Recognizing these genetic factors is essential for advancing personalized strategies in MASLD and CVD prevention and treatment.

Available evidence has also shown that alterations of specific microRNAs can cause lipid accumulation in hepatocytes and increase the risk of fibrosis and necrosis [162,163]. MiR-34a is often found to be upregulated in individuals with MASLD and MASH. Its increased expression contributes to liver damage by promoting apoptosis, inflammation, and the accumulation of lipids within liver cells, due to its ability to suppress essential regulators of lipid metabolism and cellular stress responses, such as SIRT1 and PPARα [164]. MiR-21 is also upregulated in MASH and is key in driving disease progression, enhancing fibrogenesis and inflammation by activating signaling pathways such as TGF-β and PTEN/AKT [165]. In contrast, miR-122, the most abundant liver-specific miRNA with a protective role in maintaining hepatic homeostasis, tends to be downregulated. This is associated with the impairment of normal lipid metabolism and the promotion of liver inflammation and fibrosis, highlighting its protective role in maintaining hepatic homeostasis [166].

### 3.2. Clinical Aspects

In 2021, for the first time, the European Society of Cardiology (ESC) included MASLD as an independent, often unappreciated, cardiovascular risk factor [167], due to the evidence that CVDs are the leading cause of death in patients with MASLD [168]. The high prevalence of cardiovascular disease in patients with MASLD is the result of a complex interaction between hepatic steatosis and classic and non-classic risk factors such as obesity, hypertension, T2M, insulin resistance, proinflammatory mediators, pro-atherogenic dyslipidemia, oxidative stress, endothelial dysfunction, and platelet activation [169]. Different studies have shown that the degree of liver fibrosis is a major predictor not only of the severity of liver disease but also of the risk of major cardiovascular events [170].

MASLD is closely linked to various cardiovascular complications, largely as a result of its underlying metabolic disturbances (Figure 1).

Impaired hepatic metabolism leads to excess lipoprotein production and diminished clearance. Accumulation with activation of hormone-sensitive lipases, which induce hypertriglyceridemia, increases VLDL, apolipoprotein B, and very low density lipoprotein and reduces HDL [124,171], causing elevated total and LDL cholesterol levels and contributing to atherosclerotic plaque development [61,62]. Excess caloric intake is also converted into triglycerides, which stimulate the production of small dense LDL (sdLDL) particles and contribute to arterial wall thickening, thereby elevating cardiovascular risk [172]. In MASLD, HDL cholesterol (HDL-C), normally protective through reverse cholesterol transport, is often reduced due to disruptions in HDL metabolism involving enzymes such as LCAT and CETP [173]. This lipid triad, characterized by high triglycerides, low HDL-C, and sdLDL combined with MASLD’s proinflammatory and insulin-resistant state, significantly increases the risk of ASCVD and cardiovascular events.

In individuals with MASLD, cardiovascular events such as coronary artery disease (angina and myocardial infarction) are mainly driven by accelerated atherosclerosis and lipid buildup within the arterial walls. Evidence shows that individuals with both myocardial infarction and MASLD have a significantly higher mortality risk than those with myocardial infarction alone, suggesting that MASLD may be an independent risk factor for coronary heart disease [174]. Moreover, ischemic cardiomyopathy can amplify systemic inflammation and upregulate profibrotic mediators, potentially worsening hepatic fibrosis in MASLD through inter-organ signaling pathways [175]. A systematic review and meta-analysis found that MASLD increases the risk of coronary artery calcium (CAC) progression [176], with further studies showing a stronger association in MASLD patients [177], suggesting a heightened risk of coronary artery disease. The link between MASLD and ASCVD is further supported by evidence showing an increased risk of ischemic stroke [178].

MASDL is also associated with several subclinical markers of peripheral artery disease. A correlation has been shown between hepatic steatosis and increased carotid intima–media thickness (CIMT), coronary artery calcifications, and high-risk and unstable obstructive plaques [169]. A large meta-analysis involving 85,395 participants, including 29,493 with MASLD, showed an increased risk of subclinical atherosclerosis compared to individuals without MASLD, with subgroup analyses showing greater CIMT, arterial stiffness, coronary artery calcification, and endothelial dysfunction, measured by flow-mediated dilation [102]. CIMT correlates with the severity of MASH, extending this association to the severity of histological features within MASLD and MASH [101]. MASH involves the endothelial layer and the smooth muscle cells, with a dysregulation of the vascular tone and myocardial blood flow, reduced coronary artery flow reserve, and compromised collateral vessel formation in response to ischemia, ultimately resulting in myocardial ischemia [104].

MASLD is also associated with a relevant cardiovascular risk factor, such as hypertension, in a bidirectional relationship, with each condition contributing to the other’s progression [179]. Insulin resistance, a key feature of MASLD, can increase sympathetic activity and promote renal salt and water retention, leading to elevated blood pressure [180]. Chronic inflammation in MASLD further contributes to vasoconstriction and hypertension [181]. Preclinical studies suggest that adipose-derived angiotensinogen plays a role in blood pressure regulation [182], and in humans, a genetic variant in the *AGTR1* gene (rs5186 A1166C) is linked to hypertension in MASLD patients [183]. These observations suggest that MASLD may activate the RAAS, contributing in turn to elevated vascular resistance and blood volume. Consequently, a comprehensive approach to managing both MASLD and hypertension is recommended to reduce the additive CVD risk.

Moreover, a dysregulation of the renin–angiotensin–aldosterone system (RAAS) has been documented in patients with MASLD, which plays a role in the development of hypertension, hypertension-mediated organ damage, increased vascular resistance, and cardiac remodeling [184]. Ectopic fat accumulation in the liver also promotes endothelial dysfunction and increases sympathetic tone, leading to arterial stiffening [185].

A greater prevalence of left ventricular diastolic dysfunction and cardiac remodeling in MASLD patients has been described, especially those with overweight or diabetes and hypertension, pointing to a possible connection with heart failure [186]. A 14-year large Chinese cohort study confirmed a higher incidence of heart failure in MASLD, particularly among individuals under 45 years, indicating increased risk in younger populations [187,188].

**Figure 2:**
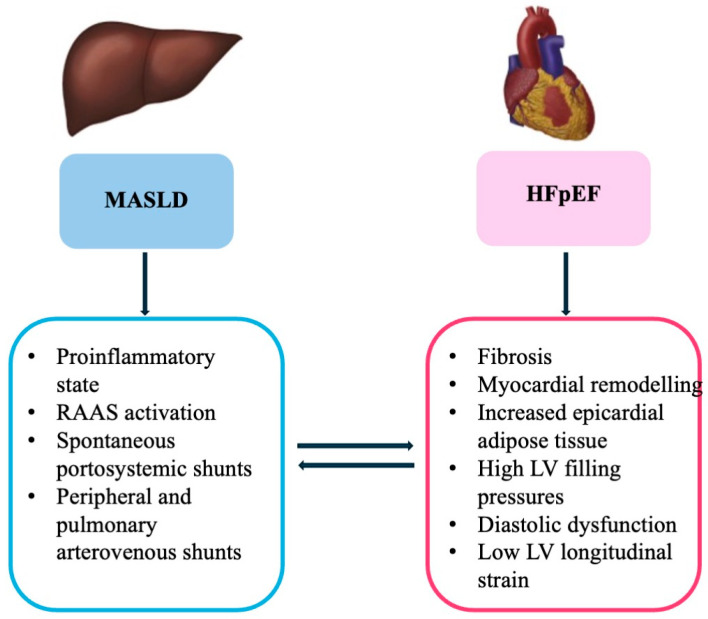
MASLD and HFpEF interrelation. MASLD, metabolic-associated steatotic liver diseases); HFpEF, heart failure with preserved ejection fraction).

A bidirectional relationship exists between MASLD and heart failure (HF), in particular, with preserved ejection fraction (HFpEF), in a self-perpetuating cycle, where the worsening of one condition often accelerates the progression of the other [8]. Indeed, MASLD and HFpEF share common risk factors, including obesity, diabetes, and metabolic dysfunction (Figure 2).

In a study including 26,676 patients, MASLD was associated with a 2.5-fold-higher risk of new-onset HF and a 2.30-fold-higher risk for HF-related hospitalizations, with a further risk increase in patients with more cardiometabolic risk factors [106].

Consistently, a meta-analysis including 11,242,231 middle-aged individuals showed that NAFLD was associated with a 1.5-fold-higher risk of new-onset HF independently from age, sex, ethnicity, adiposity measures, diabetes, hypertension, and other common cardiovascular risk factors [189].

Progressive cardiac remodeling, including changes in left ventricular geometry, altered strain patterns, enhanced epicardial fat thickness, and impaired diastolic function, has been documented in MASLD [189]. In addition, the excess fatty acids contribute to cardiac lipotoxicity, impairing myocardial energy metabolism and function [190].

The progression of hepatic fibrosis also contributes to complex hemodynamic alterations, starting from increased sinusoidal resistance; the progressive obstruction to transhepatic blood flow; portal hypertension; and subsequent hemodynamic adaptations, including the development of spontaneous portosystemic shunts [191,192]. Consequently, vasoactive mediators from the splanchnic circulation bypass hepatic first-pass metabolism, resulting in pulmonary and systemic vasoconstriction and cardiovascular remodeling [193]. As a response to arterial stiffness and increased afterload, concentric left-ventricular remodeling occurs, with reduced left ventricular compliance, impaired relaxation, and arterial–ventricular coupling [194].

Moreover, MASLD has been associated with a greater risk of both prevalent and incident atrial fibrillation (AF) [195]. A meta-analysis found a higher incidence of atrial fibrillation (AF) in middle-aged and elderly MASLD patients, particularly those with diabetes [196]. However, subsequent studies suggest that liver fibrosis, indicated by increased stiffness rather than steatosis itself, is more closely linked to AF risk, even in the absence of fatty liver [197]. These findings highlight the intricate relationship between liver disease and cardiac arrhythmias, reinforcing the need to incorporate MASLD into cardiovascular risk assessment and screening efforts.

In patients with MASLD, the altered metabolic profile and insulin resistance are also involved in the development of renal damage, accounting for a two-fold increased risk of terminal kidney disease even in the absence of pre-existing chronic kidney disease [198,199,200].

## 4. Assessment of CVD Risk in MASLD Patients

The strong association between MASLD and cardiometabolic risk factors, such as obesity, T2DM, hypertension, and dyslipidemia, necessitates comprehensive cardiovascular evaluation. Given the global burden of MASLD and the limitations of liver biopsy, the development of reliable noninvasive tools for diagnosis, staging, and prognostication is crucial. Traditional risk assessment tools, including the Framingham Risk Score and ASCVD calculator, may underestimate cardiovascular risk in MASLD, especially in younger or asymptomatic individuals. Noninvasive fibrosis scoring systems (e.g., FIB-4 and NAFLD Fibrosis Score) and noninvasive imaging techniques provide valuable prognostic information, as advanced hepatic fibrosis has been independently associated with increased cardiovascular events [115,201,202]

Several scoring systems based on routine clinical and laboratory parameters have been validated to estimate the presence of hepatic fibrosis. The FIB-4 index, NAFLD Fibrosis Score (NFS), and AST to Platelet Ratio Index (APRI) are noninvasive tools that classify patients into low, intermediate, or high risk for advanced liver fibrosis. They are especially useful in primary care and metabolic clinics to support referral decisions and minimize the need for liver biopsy. NFS is a widely used noninvasive tool for estimating the degree of hepatic fibrosis in patients with MASLD. Studies have shown that elevated NFS values are associated with an increased risk of major adverse cardiovascular events (MACEs). Specifically, compared to individuals in the low-risk NFS group, those in the intermediate group had a hazard ratio (HR) of 1.938 for MACEs, while those in the high-risk group had an HR of 3.492 [203]. However, NFS has limitations, including a tendency to overestimate fibrosis in morbidly obese patients and a suboptimal reliability compared to other methods, like elastography. The FIB-4 index (initially developed for HCV patients) is well validated; simple to use; and correlates with advanced fibrosis, systemic inflammation, and increased prothrombotic activity. Notably, FIB-4 is also an independent predictor of MACEs in individuals with MASLD [204]. APRI has shown limited accuracy in detecting fibrosis in MASLD patients [205]. Among these commonly used scoring systems, the FIB-4 index remains the most accurate and reliable for prognosticating liver-related and cardiovascular outcomes. However, its accuracy can be affected by age, other liver diseases, and conditions that influence AST, ALT, or platelet counts [206].

Additional fibrosis markers, such as the Forns index and Hepamet Fibrosis Score (HFS), have also demonstrated strong performance in ruling out advanced fibrosis and show positive correlations with cardiovascular risk, thus offering valuable tools for clinical risk stratification in chronic liver disease [207].

Imaging-based methods like transient elastography and acoustic radiation force impulse (ARFI) imaging offer noninvasive, rapid, and patient-friendly alternatives to liver biopsy for assessing liver stiffness. ARFI provides quantitative stiffness measurements through elastograms that map fibrosis across the liver and is particularly effective in detecting significant fibrosis and cirrhosis [208,209]. Increased stiffness correlates with subclinical atherosclerosis and arterial stiffness, making these tools valuable for monitoring disease progression and treatment response in chronic liver diseases, including MASLD, where fibrosis indicates disease severity and cardiovascular risk [210]. However, its accuracy may be affected by obesity and liver inflammation. Further research is needed to enhance ARFI, integrate it with biomarkers, and validate its role in broader liver and cardiovascular risk assessment.

More recently, magnetic resonance elastography (MRE) has emerged as a highly accurate modality for quantifying hepatic fibrosis [211] and is also associated with systemic inflammation and endothelial dysfunction, both key drivers of cardiovascular pathology. Magnetic resonance elastography (MRE) has demonstrated superior sensitivity and accuracy compared to transient elastography (TE) in detecting significant liver fibrosis and cirrhosis [212]. However, Magnetic resonance elastography (MRE) is more sensitive and accurate than transient elastography (TE) for detecting significant fibrosis and cirrhosis [212], but high costs and specialized equipment requirements limit its use.

Magnetic resonance spectroscopy (MRS) provides precise quantification of hepatic fat and metabolic activity, making it a valuable tool for assessing fatty liver disease [213] and supporting cardiovascular risk evaluation, particularly in individuals with insulin resistance or dyslipidemia. Beyond liver assessment, MRS can detect metabolic abnormalities, such as elevated triglycerides and lactate, that indicate myocardial stress and potential ischemia. It can also identify lipid-rich atherosclerotic plaques within the arterial vasculature, which are key contributors to coronary artery disease and cerebrovascular events [214].

Combining MRS with MRE enables simultaneous assessment of liver steatosis and fibrosis, providing a more comprehensive evaluation of liver health and improving its usefulness in studying MASLD and related cardiovascular risk.

Emerging imaging biomarkers like the pericoronary fat attenuation index (FAI), obtained through coronary computed tomography angiography (CCTA), offer additional insight into cardiovascular risk. FAI indicates pericoronary inflammation and is notably higher in MASLD patients. Elevated FAI levels have been linked to worse cardiovascular outcomes [215,216], highlighting vascular inflammation as a potential driver of CVD progression in this population. Adjunctive cardiovascular evaluations, including CAC scoring, carotid intima–media thickness (cIMT), and biomarkers such as high sensitivity C-reactive protein (hs-CRP), NT-proBNP, and high-sensitivity troponins, enhance early detection of subclinical disease.

In addition to clinical scoring systems, circulating biomarkers, including cytokeratin-18 (CK-18) fragments, procollagen III N-terminal peptide (PIIINP), hyaluronic acid, and TIMP-1, have been investigated for their utility in detecting steatohepatitis and liver fibrosis. Composite panels such as FibroTest, Enhanced Liver Fibrosis (ELF) score, and FAST score (combining FibroScan and AST) have also shown promise in identifying patients at risk for progressive disease. Emerging biomarkers, including microRNAs, extracellular vesicles, and metabolomic/lipidomic signatures, are under active investigation to improve diagnostic accuracy and predict cardiovascular outcomes. Integrating these biomarkers with imaging modalities like transient elastography or magnetic resonance techniques may offer a more comprehensive, noninvasive approach to evaluating MASLD severity and related cardiometabolic risk [202,217,218,219].

## 5. Therapeutic Perspectives

MASLD markedly elevates the risk of CVD in affected individuals. As a result, clinicians need to implement targeted management and preventive strategies to mitigate cardiovascular risk in this patient population. A multidisciplinary approach integrating hepatology, cardiology, and endocrinology is relevant for optimal CVD screening, prevention, and management in MASLD patients [202,204,205].

### 5.1. Non-Pharmacologic Interventions

Caloric restriction and physical exercise are fundamental in managing MASLD; improving metabolic homeostasis, lipid metabolism, and insulin resistance; and increasing energy expenditure [220]. Frequent rapid eating increases the risk of MASLD in both men and women. At the same time, brisk walking appears to offer protective effects, especially in women [221], emphasizing the importance of lifestyle modifications in preventing MASLD. Additionally, weight reduction, particularly through a combined healthy diet and regular physical exercise, can significantly improve liver health and lower key cardiovascular risk factors, such as hyperlipidemia and hypertension.

Effective management of MASLD requires regular monitoring and treatment of cardiovascular risk factors such as obesity, hypertension, dyslipidemia, and diabetes. In overweight or obese patients, a 7–10% weight loss is recommended to reduce liver fat and improve metabolic and vascular outcomes [222]. Dietary approaches, including low-carbohydrate, ketogenic, low-fat, and high-protein Mediterranean diets, have reduced liver steatosis and associated comorbidities [223]. Smoking, a risk factor for both MASLD and CVD, should be targeted through cessation [224]. Bariatric surgery, when indicated, supports substantial weight loss, improves metabolism via gut hormone modulation, and has been shown to reverse liver damage and lower cardiovascular risk in MASLD and MASH patients [225].

### 5.2. Pharmacologic Intervention

Different drugs with metabolic and cardiovascular effects have been tested (Table 3). Metformin, a biguanide drug currently used in treating T2DM due to its efficacy in reducing endogenous glucose production, has shown controversial evidence in MASLD experimental models and clinical studies [226]. The administration of 300 mg/kg/day metformin has been associated with a reduction of the incidence of MASLD in C57Bl/6J mice [227]. In diabetic patients with MASLD, metformin administered for 52 weeks at high dosages reduced hepatic steatosis [228]. In another study, metformin treatment has also been demonstrated to decrease the levels of inflammatory markers such as hs-CRP and ferritin [229]. The oral administration of metformin at high dose combined with a low dose (mean 26 mg/day) of pioglitazone for one-year improved liver steatosis, inflammation, and insulin resistance parameters in diabetic subjects with MASLD [230]. On the other hand, a 24-week prospective randomized placebo-controlled trial showed that metformin did not produce additive effects on hepatocyte lipid reduction in combination with type 2 sodium glucose transporter inhibitors (SGLT2is) and glucagon-like peptide-1 receptor agonists (GLP1-RAs) [231].

Thiazolidinediones (TZDs) are PPARγ agonists used in T2DM management, which have shown potential to reduce liver steatosis in MASLD and improve cardiovascular risk factors [232]. By enhancing insulin sensitivity, they lower hepatic fat and support metabolic health. The oral administration of pioglitazone at a dose of 30 mg/day has been demonstrated to improve liver metabolic, fibrosis, and other histological parameters in MASH patients without diabetes [233]. However, pioglitazone should be prescribed with caution in patients with obesity, diastolic dysfunction, and heart failure since it has been associated with an increased risk of mortality and rehospitalizations. It has been suggested that a novel dual PPARα/γ agonist (G4/G5) may offer greater benefits by reducing hepatic fat, improving insulin sensitivity, and minimizing side effects like weight gain and fluid retention, with a better safety profile than traditional TZDs [234].

SGLT2i and GLP1-R agonists are antidiabetic agents gaining an emerging role in managing MASLD, with an increasing body of evidence from both experimental and clinical studies in reducing hepatic steatosis and lowering associated cardiovascular risk.

In rodent models of MASLD, SGLT2i downregulated the expression of lipid synthesis genes and reduced lipid accumulation in the liver and inflammatory factors [235]. In preclinical models SGLT2i therapy is associated with a reduction in the expression of novel lipogenic genes, such as fatty acid synthase (Fasn), stearoyl-CoA desaturase 1 (Scd1), and acetyl-CoA carboxylase 1 (Acc1), reducing lipid synthesis and hepatic lipidosis [181]. In addition, the increase in serum glucagon levels associated with SGLT2i treatment leads to β-oxidation stimulation, to the upregulation of antioxidant systems, and the decrease of free radical species and circulating free fatty acid, thus resulting in the improvement of liver fat accumulation and hepatic lipotoxicity [236]. A randomized controlled trial showed that the SGLT-2 inhibitor empagliflozin significantly reduced liver fat content in non-diabetic MASLD patients over 52 weeks [237]. Empagliflozin has also been shown to promote the SIRT-1/PGC-α/PPAR-α pathway, which enhances fatty acid β-oxidation and lipid catabolism, and to decrease the expression of lipogenic genes *PPAR-γ*, *SREBP1c*, and *FAS* [238]. In addition, empagliflozin has been demonstrated to enhance autophagy of hepatic macrophages via the AMPK/mTOR signaling pathway [239]. Moreover, empagliflozin has been also shown to protect against MASLD through the inhibition of inositol requiring enzyme 1 (IRE1a) and X-box binding protein 1 (Xbp1); activating transcription factor 4 (ATF4) and C/EBP homologous protein (CHOP); and activating transcription factor 6 (ATF6), involved in endoplasmic reticulum stress induced inflammation and apoptosis of hepatic cells [181]. Consistently, dapagliflozin treatment significantly suppressed oleic acid (OA)-induced lipid accumulation in L02 cells through increased fatty acid oxidation, with detected elevated levels of PGC-1 and the activation of the AMPK/mTOR pathway [179]. Canagliflozin-treated HepG2 cells demonstrated increased expression of the cell growth regulator hepatocyte nuclear factor 4 (HNF4), with a reduced expression of cyclin D1, cyclin D2, and cdk4, resulting in cell cycle arrest in a hepatocellular carcinoma (HCC) cell line [180].

SGLT2is have been demonstrated to reduce the production of inflammatory cytokines, such as TNF-α, MCP-1, IL-1β, and IL-6, and to attenuate the activation of the NLRP3 inflammasome in MASLD animal models [182]. In addition, a decrease in serum caspase-3 levels and an increased expression of hepatic Bcl-2 have been detected, attenuating liver injury [183]. In a rodent model, treatment with low-dose (1 mg/kg/day) dapagliflozin and ipragliflozin for 12 weeks reduced the expression of the pro-inflammatory markers Emr1 and Itgax [240]. In a meta-analysis including 699 patients, SGLT2i significantly improved indicators of liver fibrosis, including liver stiffness measurement (LSM), controlled attenuation parameter (CAP), serum ferritin, serum type 4 collagen 7s, and FIB-4 index [241]. In other studies, scores of steatosis, lobular inflammation, ballooning, and fibrosis stage decreased after 5-year treatment with SGLT2i [242].

Recent preclinical studies in MASLD and MASH patients have demonstrated that GLP-1 receptor agonists (GLP-1 RAs) may improve metabolic profiles, liver steatosis as a consequence of indirect inhibition of hepatic gluconeogenesis via the entero-pancreas-liver axis and a beneficial role on liver lipid metabolism [24,25], and may help reduce cardiovascular risk. Experimental studies suggest that GLP-1 RAs reduce hepatic triglyceride accumulation by activating AMPK, stimulating fatty acid oxidation, and inhibiting lipogenesis [104]. Additionally, GLP-1 reduces pro-inflammatory cytokine expression and indirectly modulates hepatic stellate cell activation, a key step in developing liver fibrosis [104]. Semaglutide, a GLP-1R agonist, improves gut microbiota, lipid profiles, and glucose metabolism, significantly improving MASLD and supporting its potential as a treatment for hepatic steatosis [243]. In another study including 396 subjects treated with GLP-1R agonists, significant reductions in the liver fat content waist circumference, g-glutamyl transferase, and hemoglobin were registered [244]. In a meta-analysis conducted on patients with biopsy-proven MASH and fibrosis, GLP-1R agonists were associated with a significantly higher incidence of MASH resolution compared to placebo [245]. A systematic review including 6313 participants confirmed the efficacy of GLP-1R agonists on hepatic steatosis and inflammation [246]. However, no sufficient data are available about the potential benefits of GLP-1R agonists on the regression of fibrosis and the prevention of the progression of steatosis to MASH and cirrhosis [247].

Another class of drugs with potential effects on MASLD is represented by tirzepatide, which combines the action of both glucose-dependent insulinotropic polypeptide (GIP) and GLP-1 receptor agonist [248]. This class of drugs in commonly used for glycemic control in T2DM patients. In the SYNERGY-NASH phase 2 trial, tirzepatide significantly reduced MASH biomarkers (AST/ALT, keratin-18 M30, and procollagen III) and c-reactive protein in diabetic patients. After 52 weeks, tirzepatide was more effective than placebo in achieving MASH resolution without worsening fibrosis [249].

Furthermore, a phase 2 trial showed that survodutide, a dual GLP-1 and glucagon receptor agonist, improved MASH without aggravating fibrosis, highlighting its potential as a future therapy [245].

Inflammation is central to MASLD pathogenesis, making its pharmacological control a key therapeutic target. Anti-inflammatory agents such as NSAIDs, corticosteroids, statins, and pentoxifylline have shown a potential role in reducing hepatic inflammation and slowing disease progression. Aspirin may also reduce fibrosis progression in MASLD patients [250,251]. Given MASLD’s association with atrial fibrillation (AF) and prothrombotic states, antithrombotic therapies, including aspirin and oral anticoagulants, may benefit patients by lowering cardiovascular risk and potentially attenuating liver fibrosis [252].

Obeticholic acid (OCA), a farnesol X receptor (FXR) agonist, has been associated with favorable effects in MASLD subjects, improving glycolipid metabolism, insulin sensitivity, and mitochondrial fatty acid oxidation, and reducing liver inflammation and fibrosis [245]. OCA treatment also decreased fibrosis in MASH patients. However, long-term OCA treatment has been shown to increase the risk of skin and subcutaneous tissue diseases and gastrointestinal disorders, thus limiting its use in clinical practice [253].

Resmetirom, a thyroid hormone β-receptor agonist, effectively reduces liver fat, improves liver histology (MASH resolution and fibrosis), and lowers liver damage biomarkers without significantly impacting body weight or glucose metabolism [254]. In the phase 2 ENLIVEN trial, pegozafermin (an FGF21 analogue) significantly improved liver fibrosis without worsening MASH in biopsy-confirmed cases, supporting its progression to phase 3 trials [255].

Dyslipidemia, a major contributor to MASLD and CVD, is effectively managed with statins, which remain the most studied and widely used lipid-lowering agents. Different studies have suggested that statin therapy might improve steatosis and fibrosis in MASLD patients, also reducing the risk of cirrhotic decompensation and mortality in patients with cirrhosis. Statins have been associated with the reduction of cardiovascular risk in patients with MASLD/MASH [256]. Ezetimibe has also improved liver fibrosis scores and histology in MASLD patients [257]. PCSK9 inhibitors have been shown to reduce fat accumulation in the liver, but not the risk of progression to fibrosis [258].

Despite potential elevations in transaminases, statins should not be discontinued in MASLD patients, as they offer anti-inflammatory, antioxidant, antifibrotic, and plaque-stabilizing benefits, with proven safety in this population [252,259].

Emerging evidence links MASLD with an increased risk of prehypertension and hypertension, especially in advanced stages, indicating that MASLD may act as an independent risk factor for elevated blood pressure [260,261]. It can also contribute to early-onset hypertension, even in the absence of other metabolic abnormalities. Managing blood pressure, particularly in non-obese hypertensive individuals, may help prevent or mitigate MASLD progression [262]. While beta-blockers may blunt hypoglycemic responses in MASLD patients with T2DM, angiotensin receptor blockers (ARBs) and ACE inhibitors (ACEIs) show promise due to their anti-inflammatory and antifibrotic properties, as the RAAS pathway is involved in both MASLD and CVD. However, their use specifically for fibrosis control in MASLD should be cautiously approached and further validated [263].

## 6. Knowledge Gaps and Research Priorities in MASLD and Cardiometabolic Disease

Despite significant advances in the understanding of MASLD, important uncertainties remain that hinder the translation of this knowledge into effective strategies for cardiovascular risk reduction. These gaps span the domains of pathophysiology, diagnosis, and treatment, and addressing them is essential to improve integrated care for patients with MASLD and cardiometabolic disease.

From a pathophysiological standpoint, it remains unclear to what extent hepatic inflammation, fibrosis, ectopic fat depots, and hepatokine release directly contribute to cardiovascular disease, independently of obesity, type 2 diabetes, and hypertension [38,39,40,41,106,148,149,150,151,152,153,154,155,156,157,158,159,160]. While epidemiological evidence strongly supports associations between MASLD and atherosclerotic cardiovascular disease, heart failure (particularly with preserved ejection fraction) atrial fibrillation, chronic kidney disease, and thromboembolic events [189,190,191,192,193,194,195,196,197,198,199,200], the precise causal pathways remain incompletely defined. Systemic inflammation, endothelial dysfunction, oxidative stress, and platelet activation are likely central mediators [106,107,108,109,110,111,112,113,114,115,116,117,118,119,120], yet their relative contributions and interactions require further elucidation through integrative multi-omics and longitudinal mediation studies. Similarly, the role of the gut–liver–heart axis, encompassing microbiota dysbiosis, bile acid signaling (FXR/FGF19), and microbial metabolites, is increasingly recognized but remains insufficiently explored in interventional studies [132,133,134,135,136,137,138,139,140,141,142,143,144]. Genetic variants such as *PNPLA3* and *TM6SF2* influence hepatic outcomes, but their net effects on cardiovascular risk and responsiveness to therapy are uncertain [148,149,150,151,152,153,154,155,156,157,158,159,160,161]. In addition, the heterogeneity of MASLD across the life course, including sex-specific patterns, the “lean” phenotype, and early-onset disease, warrants further study to clarify their implications for cardiovascular trajectories [82,83,84,85,167,168,169,170].

In the diagnostic field, conventional cardiovascular risk scores such as the Framingham Risk Score or ASCVD calculator may underestimate cardiovascular risk in MASLD [201,202,203]. Recalibrated or disease-specific models incorporating measures of hepatic fibrosis and metabolic dysfunction are lacking. The optimal sequencing and thresholds for noninvasive tests (e.g., FIB-4, NAFLD fibrosis score, elastography, and magnetic resonance elastography) in predicting cardiovascular, as well as hepatic, outcomes remain to be determined [204,205,206,207,208]. Multimodality imaging approaches, including coronary artery calcium scoring, carotid intima–media thickness, and pericoronary fat attenuation index, show promise for refining risk prediction but require prospective validation in MASLD populations [102,176,177,178]. Several biomarkers, such as ELF and FAST scores, cytokeratin-18, fibrosis-related peptides, extracellular vesicles, and circulating microRNAs, have demonstrated potential but need standardization, comparative evaluation, and integration with imaging modalities to improve prediction of cardiovascular endpoints [162,163,164,165,166]. Moreover, the optimal screening strategies, including timing, patient selection, and cost-effectiveness in MASLD, have yet to be established [36,37,201,202].

In terms of treatment, multiple drug classes, including GLP-1 receptor agonists; SGLT2 inhibitors; dual GLP-1/GIP agonists such as tirzepatide; and emerging agents like thyroid hormone receptor-β agonists, FGF21 analogues, and FXR agonists, have shown beneficial effects on hepatic and metabolic parameters [245,246,247,248,249,250,251,252,253,254,255]. However, evidence for their direct impact on major adverse cardiovascular events, heart failure hospitalizations, arrhythmias, and cardiovascular mortality in MASLD-enriched populations is limited [167,168,169,170,189,190,191,192,193,194,195,196,197,198,199,200]. Similarly, although antifibrotic agents can induce histological regression, their capacity to reduce cardiovascular risk remains to be proven. The optimal management of blood pressure, the use of RAAS blockade, and the role of antiplatelet or anticoagulant therapy in MASLD patients with a prothrombotic phenotype require randomized clinical trial data that balance cardiovascular benefit with hepatic safety [250,251,252,253,254,255,256,257,258]. While statins are generally safe and effective for reducing atherosclerotic cardiovascular risk in MASLD, evidence for PCSK9 inhibitors, ezetimibe, and combination lipid-lowering strategies in improving both hepatic and cardiovascular outcomes is still sparse. Lifestyle interventions, particularly those achieving ≥ 7–10% sustained weight loss, are effective in improving hepatic and cardiometabolic health, but long-term durability, especially following bariatric or metabolic surgery, needs to be demonstrated in pragmatic trials [76,77,78,79,80,136,137,138,139,140,141,142,143,144]. Finally, there is a lack of implementation studies evaluating multidisciplinary, pathway-driven models of care integrating hepatology, cardiology, and endocrinology, as well as using digital algorithms for noninvasive-test-first triage and risk-based referral.

Addressing these knowledge gaps will require the creation of large, prospective MASLD cohorts with adjudicated cardiovascular outcomes, serial liver–cardiovascular phenotyping, and biobanked biospecimens [38,39,40,41,148,149,150,151,152,153,154,155,156,157,158,159,160,161,189,190,191,192,193,194,195,196,197,198,199,200]. Randomized controlled trials powered for both hepatic and cardiovascular co-primary endpoints are needed to test pharmacologic and lifestyle interventions [245,246,247,248,249,250,251,252,253,254,255]. In parallel, validated, implementable risk algorithms that combine noninvasive tests, imaging, and biomarkers should be developed to guide cardiovascular screening and therapy in MASLD [201,202,203,204,205,206,207]. Such integrated, multidisciplinary research efforts are essential to fully characterize the cardiohepatic continuum and improve outcomes in this growing patient population.

## 7. Conclusions

MASLD is a highly prevalent yet under-recognized and underdiagnosed condition with clinical consequences extending far beyond the liver. Robust clinical and experimental evidence identifies MASLD as a systemic disorder closely intertwined with cardiometabolic health. The associations with obesity, T2DM, hypertension, and dyslipidemia reflect a complex interplay of different conditions, including insulin resistance, chronic inflammation, oxidative stress, lipid abnormalities, the influence of genetic variants, and gut microbiota dysbiosis, exerting adverse effects on multiple organs, including the cardiovascular system, and substantially increasing cardiovascular risk.

MASH represents the progressive, inflammatory–fibrotic end of the MASLD spectrum and carries a heightened risk of cirrhosis and hepatocellular carcinoma. Crucially, it is also associated with increased cardiovascular morbidity and mortality. The strong, often bidirectional relation between MASLD/MASH and cardiometabolic disorders suggests the need for integrating MASLD into established cardiovascular risk assessment models.

A multidisciplinary strategy that enables prompt detection, comprehensive risk profiling, and coordinated therapeutic interventions targeting both liver and heart is necessary to enable timely screenings and interventions and reduce the MASLD burden in at-risk populations. Widely available noninvasive tools, such as the FIB-4 index, elastography, and MRI-based imaging, provide reliable means for dual risk stratification of hepatic and cardiovascular complications.

Clinical, biochemical, and genetic markers, including *PNPLA3* and *TM6SF2* variants, promise to predict disease progression and guide individualized treatment decisions.

Precision medicine might contribute to better identifying the cardiometabolic causes and consequences of MASLD and improve risk prediction, prevention, and treatment. In addition, the response to emerging MASLD therapeutics might be predicted based on clinical, laboratory, and genetic analyses, providing further guidance to MASLD management [264].

While lifestyle modification remains the foundation of therapy, several pharmacological agents, such as GLP-1 receptor agonists, dual GIP/GLP-1 agonists, SGLT2 inhibitors, and statins, have demonstrated dual benefits for hepatic outcomes and cardiometabolic risk reduction. In addition, novel agents targeting inflammation, fibrosis, and metabolic dysfunction are under evaluation to decrease liver-related and cardiovascular mortality in high-risk patients.

In this regard, this narrative review provides novel perspectives on MASLD and its cardiovascular implications by achieving the following:

- Reinforcing MASLD as a systemic disorder with strong bidirectional links to cardiovascular disease, supporting its inclusion in cardiovascular risk assessment models.
- Highlighting gut microbiota dysbiosis as an emerging mechanistic link and therapeutic target for concurrent hepatic and cardiovascular risk reduction.
- Identifying noninvasive fibrosis assessment tools as dual predictors of hepatic and cardiovascular outcomes, enabling integrated risk management.
- Summarizing robust evidence for GLP-1 receptor agonists, SGLT2 inhibitors, and dual GIP/GLP-1 agonists in improving hepatic outcomes and cardiometabolic profiles.
- Underlining the influence of genetic variants (*PNPLA3* and *TM6SF2*) on disease progression and advocating for personalized risk stratification and treatment approaches.

## Figures and Tables

**Table 1:** Diagnostic criteria for MASLD in the presence of hepatic steatosis [7]. Diagnosis requires evidence of hepatic steatosis plus at least one of the following cardiometabolic risk factors.

Obesity or overweight, defined as BMI ≥ 25 kg/m^2^
Type 2 diabetes
Evidence of metabolic dysregulation, defined by the presence of at least one of the following, regardless of BMI: - Abdominal obesity waist circumference ≥ 102 cm in men or ≥88 cm in women; - Blood pressure ≥ 130/85 mmHg or antihypertensive treatment; - Prediabetes, defined as fasting glucose level 100–12.5 mg/dl, or 2 h after glucose load 140–199 mg/dl or glycated hemoglobin level 5.7–6.4%; - Insulin resistance (HOMA-IR) ≥ 2.5; - Serum triglyceride level ≥ 150 mg/dl or hypolipidemic treatment; - High-density lipoprotein cholesterol (HDL-c) level < 40 mg/dl for men and <50 mg/dl for women.

**Table 2:** Main differences between MASLD and MASH.

	MASLD	MASH
Histology	Presence of hepatic steatosis (≥5% of hepatocytes) with evidence of metabolic dysfunction	A subset of MASLD where steatosis is accompanied by hepatic inflammation, ballooning due to cytoskeletal collapse (keratin-18 cleavage), apoptosis, necroptosis, and fibrosis
Pathology	Hepatic lipid accumulation due to metabolic dysfunction (insulin resistance, increased de novo lipogenesis, and impaired β-oxidation)	Lipid accumulation plus hepatocellular injury from lipotoxicity, oxidative stress, and inflammatory cascades
Lipid involved	Mild increase of triglycerides, saturated fatty acids, diacylglycerols, and ceramides	Lipotoxic species (free cholesterol, ceramides, and lysophosphatidylcholines) at higher concentrations
Insulin resistance	Drives hepatic de novo lipogenesis via SREBP-1c and ChREBP activation	Exacerbates oxidative stress and mitochondrial dysfunction
Oxidative stress	ROS generation, inflammation (TNF-α, IL-6, and CRP)	ROS, mtDNA damage, impaired oxidative phosphorylation, and high inflammatory drive: ↑ TNF-α, IL-6, IL-1β, and chemokines (CCL2 and CXCL10)
Prognosis	Can remain stable if metabolic control achieved	High risk of progression to advanced fibrosis and cirrhosis

**Table 3:** Drugs with potential beneficial effects in MASLD and MASH.

Drug	Main Mechanism of Action	Effects
Metformin	Reduction of hepatic gluconeogenesis and improvement the body’s sensitivity to insulin, particularly in muscle tissue.	- Reduction of blood sugar; - Improvement of insulin resistance; - Improvement of liver steatosis and inflammation.
SGLT2i	Inhibition of SGLT2: lower blood glucose levels by preventing the kidneys from reabsorbing glucose back into the bloodstream, leading to increased glucose excretion in the urine.	- Reduction of glucose levels; - Improvement of liver fat accumulation and hepatic lipotoxicity, attenuation of liver injury; - Reduction in the expression of novel lipogenic genes, lipid synthesis and hepatic lipidosis; - Upregulation of antioxidant systems; - Reduction of inflammatory cytokines and NLRP3 inflammasome.
GLP-1R agonists	Binding to and activating GLP-1 receptors in the body, primarily in the pancreas, brain, and gastrointestinal tract, to regulate blood sugar levels and promote weight loss.	- Improvement of glycemic control, reduction of HOMA-IR; - Weight loss and BMI reduction; - Improvement of gut microbiota and lipid profile; - Reduction of liver steatosis and hepatic histological damage.
Tirzepatide	Dual agonist for the glucagon-like peptide-1 (GLP-1) and glucose-dependent insulinotropic polypeptide (GIP) receptors.	- Improvement of glycemic control; - Weight loss; - Reduction of liver fibrosis and MASH biomarkers (AST/ALT, keratin-18 M30, and procollagen III).
Thiazolidinediones (Pioglitazone)	Binding to the PPARγ receptor, a type of nuclear receptor that regulates gene expression.	- Improvement of insulin sensitivity and glucose control; - Enhancement of glucose uptake by cells; - Suppression of the production of pro-inflammatory molecules; - Promotion of the production of anti-inflammatory molecules; - Reduction of steatosis, and hepatocyte ballooning. - Reduction of hepatic lipotoxicity.
Obeticholic acid	Highly selective agonist for the farnesoid X receptor (FXR).	- Improvement of glycolipid metabolism; - Improvement of insulin sensitivity, and mitochondrial fatty acid oxidation. - Reduction of liver inflammation and fibrosis.
